# Trends in Lung Cancer Incidence Rates, Oklahoma 2005–2010

**DOI:** 10.1371/journal.pone.0119251

**Published:** 2015-04-22

**Authors:** Dana S. Mowls, D. Robert McCaffree, Laura A. Beebe

**Affiliations:** 1 Department of Biostatistics & Epidemiology, College of Public Health, University of Oklahoma Health Sciences Center, Oklahoma City, Oklahoma, United States of America; 2 Oklahoma Tobacco Research Center, Oklahoma City, Oklahoma, United States of America; Geisel School of Medicine at Dartmouth College, UNITED STATES

## Abstract

**Purpose:**

Lung cancer is the second most frequently diagnosed cancer among men and women in the United States. With cigarette smoking causing the majority of cases, patterns in lung cancer are often monitored to understand the impact of anti-tobacco efforts. The purpose of this research was to investigate trends in lung cancer incidence rates for the period 2005–2010 in Oklahoma.

**Methods:**

Data on Oklahoma’s incident cases of lung cancer (2005–2010) were obtained from the Centers for Disease Control and Prevention WONDER system. Annual percent change (APC) was calculated by linear regression to characterize trends in lung cancer incidence rates over time for the overall population, by gender, by age group, and by age group within gender. Rates were considered to increase or decrease if the p-value for trend was <0.05.

**Results:**

From 2005 through 2010, lung cancer incidence rates declined from 81.96 to 68.19 per 100,000 population, with an APC of -3.58% (p-value: 0.0220). When subgroups were examined, declines were observed among all males (APC: -4.25%; p-value: 0.0270), males <65 years (APC: -5.32%; p-value: 0.0008), females <65 years (APC: -4.85%; p-value: 0.0044), and persons aged 55–64 years (APC: -6.38%; p-value: 0.0017).

**Conclusions:**

Declines in lung cancer incidence rates occurred during 2005–2010 among the overall population and within select demographic groups in Oklahoma. Although trends were stable for several demographic groups, rates of lung cancer incidence were lower in 2010 compared to 2005. Continued evidence-based tobacco control efforts are needed to ensure further reductions in lung cancer incidence rates in the state of Oklahoma.

## Introduction

Lung cancer is the second most frequently diagnosed cancer among men and women in the United States [1]. The national costs of lung cancer are substantial and estimated to increase 21% from 2010 ($12.1 billion) to 2020 ($14.7 billion) [2]. Cigarette smoking and secondhand smoke exposure cause the majority of lung cancer cases and an estimated 80 to 90% of lung cancer deaths [3]. Since most lung cancer cases are attributed to cigarette smoking, patterns in lung cancer are expected to reflect trends in smoking, after accounting for a lag period between 5 to 20 years [4–6].

A recent report by the Centers for Disease Control and Prevention (CDC) showed statistically significant declines in lung cancer incidence among US men and women from 2005 to 2009 [7]. Variations were observed in declines, with lung cancer incidence decreasing most rapidly among men, young adults, and in the Midwest region of the US [7]. Demographic variations in lung cancer declines may reflect differing sensitivity to changing risk exposure. For instance, lung cancer rates among young adults are hypothesized to be more responsive to recent declines in smoking prevalences than older adults [6]. Geographic variations in lung cancer trends may reflect variability in smoking prevalences that are the result of differing investments in tobacco control and prevention [8]. The findings of the CDC report on lung cancer declines are hailed as a major achievement of tobacco control, as significant reductions in lung cancer are largely attributed to a multicomponent approach to tobacco prevention and control which involves evidence-based strategies such as cessation services including state quitlines, taxing cigarettes, smoking bans, and media counter-advertising.

In Oklahoma, the prevalence of current cigarette smoking among adults decreased from nearly 29% in 2001 to 24% in 2010 with a statistically significant average annual decline of 1.3% [9]. Consistent with the pattern of cigarette smoking, per capita cigarette sales declined 34%, from 108 packs in 2001 to 71 packs in 2010 [10]. With reductions in the prevalence of cigarette smoking and per capita cigarette consumption, it is hypothesized that rates of lung cancer are declining in Oklahoma. The purpose of this research is to investigate trends in lung cancer incidence rates in Oklahoma by select demographic groups for the most recent 6-year period (2005–2010).

## Materials and Methods

This research involved the analyses of existing, publicly available data and did not require review from any Intuitional Review Board. All data analyses were conducted in 2014. Data on Oklahoma’s incident cases of lung, including bronchus, cancer diagnosed during 2005 through 2010 were obtained from the National Center for Health Statistics via the Centers for Disease Control and Prevention (CDC) WONDER system [11]. CDC WONDER is an online vital records system accessible to the public and includes cancer incidence data provided by the National Program of Cancer Registries (NPCR) and the National Cancer Institute Surveillance, Epidemiology and End Results (SEER) Program. Rates provided by CDC WONDER are age-adjusted to the 2000 US standard million population. Annual percent change (APC) was calculated by least-squares linear regression on a log-linear model to characterize trends in lung cancer incidence rates over time [12]. APCs and corresponding 95% confidence intervals were calculated for the overall population, by gender and by age group (35–44 years, 45–54 years, 55–64 years, 65–74 years, and ≥75 years). In addition, APCs were further examined by age group within gender (males <65 years, males ≥65 years, females <65 years, and females ≥65 years). Rates were considered to increase or decrease if the p-value for trend was <0.05. Analyses were conducted in SAS 9.2.

## Results

During 2005 through 2010, a total of 18,616 incident cases of lung cancer were reported among residents of Oklahoma (Table 1). More incident cases of lung cancer were reported in males than females and in persons of older age. During this time period, the average age-adjusted incidence rate for lung cancer was 77.86 per 100,000 population and greater among males than females. By age, the average age-adjusted incidence rate of lung cancer was highest among those aged ≥75 years (443.77 per 100,000) and declined with decreasing age.

**Table 1:** Age-adjusted rate of lung cancer per 100,000 population, Oklahoma 2005–2010.

	2005	2006	2007	2008	2009	2010	2005–2010	
	Rate	Rate	Rate	Rate	Rate	Rate	Rate	Total cases
Overall	81.96	82.21	81.78	80.57	73.36	68.19	77.86	18616
Males	104.24	101.35	106.83	98.54	92.03	82.81	97.36	10365
Females	65.26	68.17	63.02	66.98	58.79	56.97	63.10	8251
35–44 yrs	12.19	9.08	8.83	6.25	6.33	7.89	8.48	240
45–54 yrs	61.66	61.69	58.72	59.92	62.42	53.25	59.62	1884
55–64 yrs	195.00	186.82	174.15	170.17	144.29	144.24	167.99	4170
65–74 yrs	406.59	429.79	414.07	448.54	380.81	361.17	406.05	6398
≥75 yrs	457.23	455.68	493.10	444.03	430.11	384.04	443.77	5885
Males <65 yrs	36.62	33.31	32.48	31.54	29.67	26.92	31.65	3543
Females <65 yrs	26.65	27.16	24.30	24.02	21.52	21.69	24.13	2790
Males ≥65 yrs	571.65	571.67	620.72	561.63	523.11	469.14	551.56	6822
Females ≥65 yrs	332.10	351.71	330.72	363.92	316.43	300.85	332.47	5461

**Fig 1:**
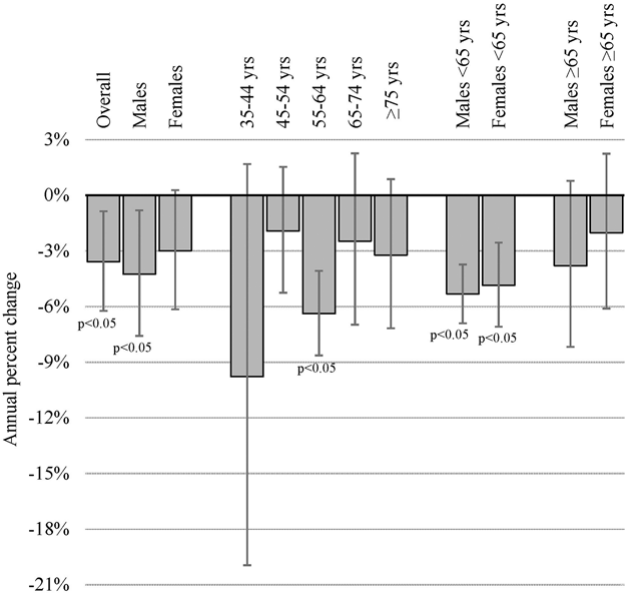
Annual percent change in lung cancer incidence rates by demographic groups—Oklahoma, 2005–2010.

In Oklahoma, lung cancer incidence rates declined overall and within select demographic groups during 2005 through 2010 (Fig 1). Overall lung cancer incidence rates declined statistically significantly from 81.96 per 100,000 population in 2005 to 68.19 per 100,000 population in 2010, with an annual percent change (APC) of -3.58% (95% Confidence Interval (CI): -6.23, -0.86; p-value: 0.0220). Lung cancer incidence rates declined among the overall male population with an APC of -4.25% (CI:-7.58, -0.81; p-value: 0.0270). Lung cancer incidence rates were stable among the overall female population (APC: -2.99%; CI:-6.15, 0.28; p-value: 0.0640). For both males and females <65 years of age, lung cancer incidence rates declined. Rates declined with an APC of -5.32% (CI:-6.90, -3.73; p-value: 0.0008) for males <65 years and an APC of -4.85% (CI:-7.09, -2.55; p-value: 0.0044) for females <65 years. Lung cancer incidence rates were stable for both males (APC:-3.80%; CI: -8.17, 0.78; p-value: 0.0819) and females ≥ 65 years (APC: -2.02%; CI:-6.11, 2.24; p-value: 0.2534).

Excluding 55–64 years, lung cancer incidence rates by age group were stable during 2005 through 2010. Among persons aged 55–64 years, lung cancer incidence rates declined with an APC of -6.38% (CI: -8.63, -4.07; p-value: 0.0017). Lung cancer incidence rates were stable among persons aged 35–44 years (APC: -9.78%; CI:-19.89, 1.56; p-value: 0.0732), 45–54 years (APC: -1.92%; CI:-5.22, 1.50; p-value: 0.1920), 65–74 years (APC: -2.47%; CI:-6.98, 2.26; p-value: 0.2166), and ≥75 years (APC: -3.23%; CI:-7.17, 0.87; p-value: 0.0929).

## Discussion

Declines in lung cancer incidence rates occurred during the period 2005 through 2010 among the overall population in Oklahoma and within select demographic groups, including males, both younger males and females, and persons aged 55 to 64 years. Although trends were stable for several demographic groups, rates of lung cancer incidence were lower in 2010 compared to 2005.

Declines in lung cancer incidence rates are attributed, in large part, to tobacco control programming. Studies have demonstrated that implementation of evidenced-based tobacco control strategies, including mass media campaigns, community programs, policy initiatives and cessation services, prevent initiation of tobacco use and promote quitting among smokers [13–15]. Moreover, previous research indicates that longer and heavier investments in comprehensive tobacco control programs are associated with greater reductions in smoking prevalences and cigarette sales [8, 16]. The state of Oklahoma has made significant strides to reduce tobacco consumption and prevalence in recent decades. For example, comprehensive, community-based, tobacco control programs have been implemented in 50 counties throughout Oklahoma and one tribal nation to counter pro-tobacco influences [17]. Although no single intervention is solely responsible for Oklahoma’s progress, these community-based best practices are associated with positive changes in key outcomes related to tobacco use, including an increase in quit attempts and Oklahoma Tobacco Helpline awareness[17].

The most dramatic reduction in lung cancer was among persons aged 35 to 44 years in Oklahoma from more than 12 per 100,000 population in 2005 to less than 8 per 100,000 population in 2010. Although non-statistically significant, this finding is consistent with results from the CDC report where US males and females aged 35 to 44 years had the most rapid decline of lung cancer incidence when compared to any other age group [7]. Patterns of lung cancer among young adults have been identified as early indicators of the impact of tobacco control. In one study, rates of lung cancer mortality among young adults from 1995–1999 were strongly and inversely correlated with state tobacco control efforts from 1992–1993 [6]. Therefore, the reduction in lung cancer incidence rates among young adults may be attributed to strategies to reduce cigarette smoking in more recent than distant years preceding case selection.

This study has a few potential limitations. First, delays in cancer reporting may result in biased incidence rates. Second, the capacity to analyze the data by further strata (e.g., gender by narrower age groups) was limited due to the small number of cases within younger age groups. Also, small sample sizes limit the ability to draw meaningful inferences for all demographic groups. The non-statistically significant pattern in lung cancer incidence rates among persons aged 35–44 years is most likely a result of insufficient power, as the average number of cases per year among this age group was small (n = 40). Or a non-significant trend may reflect insufficient lag time between tobacco control efforts and changes in lung cancer incidence rates. Regardless, stable trends in lung cancer incidence rates should be closely monitored to understand the impact of tobacco control and prevention efforts.

These findings have implications for the impact of tobacco control and prevention efforts in reducing the burden of tobacco-related disease among residents of Oklahoma. Declines in lung cancer incidence rates occurred during the period 2005 through 2010 within the overall population and among select demographic groups in Oklahoma. Although trends were stable for several demographic groups, rates of lung cancer incidence were lower in 2010 compared to 2005. Continued evidence-based tobacco prevention and control efforts are needed to ensure further reductions in lung cancer incidence rates in the state of Oklahoma.
